# Interior regularity of obstacle problems for nonlinear subelliptic systems with VMO coefficients

**DOI:** 10.1186/s13660-018-1647-5

**Published:** 2018-03-05

**Authors:** Guangwei Du, Fushan Li

**Affiliations:** School of Mathematical Sciences, Qufu Normal University, Qufu, China

**Keywords:** 35J70, 35B65, 35J50, Subelliptic systems, Obstacle problem, VMO, $L_{X,\mathrm{loc}}^{2,\lambda }$-regularity

## Abstract

This article is concerned with an obstacle problem for nonlinear subelliptic systems of second order with VMO coefficients. It is shown, based on a modification of *A*-harmonic approximation argument, that the gradient of weak solution to the corresponding obstacle problem belongs to the Morrey space $L_{X,\mathrm{loc}}^{2,\lambda }$.

## Introduction

In this paper we consider weak solutions to an obstacle problem for the following nonlinear subelliptic system in a bounded domain Ω of Euclidean space $\mathbb{R}^{n}$:

1.1 $$ X_{\alpha }^{\ast } \bigl( a_{ij}^{\alpha \beta }(x)X_{\beta }u^{j} \bigr) = {B_{i}}(x,u,Xu)+X_{\alpha }^{\ast }g_{i}^{\alpha }(x,u,Xu),\quad i = 1,2,\ldots ,N, $$

where $X = ({X_{1}},\ldots,{X_{m}})$ $(m \leq n)$ is a system of smooth real vector fields satisfying the Hörmander’s rank condition, $X_{\alpha }^{\ast }$ is the formal adjoint of ${X_{\alpha }}$.

If we set $A(x) =\{ a_{ij}^{\alpha \beta }(x)\}$, $B = ({B_{i}})$, $g = (g_{i}^{\alpha })$, then (1.1) reads

$$ -X^{\ast } \bigl( {A(x)Xu} \bigr) = B(x,u,Xu) - {X^{\ast }}g(x,u,Xu). $$

Given two vector-valued functions $\psi = ({\psi ^{1}},\ldots ,{\psi ^{N}})$ and $\theta = ({\theta ^{1}},\ldots ,{\theta ^{N}})$ with $\theta (x) \geq \psi (x)$ a.e. on *∂*Ω (i.e. ${\theta ^{i}}(x) \geq {\psi ^{i}}(x)$ a.e. on *∂*Ω, $i = 1,2, \ldots ,N$), we define the set

$$ \mathfrak{K}_{\psi }^{\theta }= \bigl\{ v \in S_{X}^{1,2} \bigl(\Omega ,\mathbb{R}^{N}\bigr):v \geq \psi \mbox{ a.e. in } \Omega ,v-\theta \in S_{X,0}^{1,2}\bigl(\Omega , \mathbb{R}^{N}\bigr) \bigr\} . $$

Here the functions *ψ* and *θ* are called obstacle and boundary datum, respectively. The function $u\in \mathfrak{K}_{\psi }^{\theta }$ is called a weak solution to the obstacle problem related to (1.1) if

1.2 $$ \int _{\Omega }{A(x)} Xu \cdot X\varphi\,dx \geq \int _{\Omega }B(x,u,Xu)\varphi \,dx+ \int _{\Omega }g(x,u,Xu)\cdot X\varphi \,dx $$

holds for all $\varphi \in C_{0}^{\infty }(\Omega ,\mathbb{R}^{N})$ with $\varphi + u\geq \psi $ a.e. $x\in \Omega $.

As we know, the uniform ellipticity requirement on coefficients is not sufficient to get the local boundedness of solutions even for one single equation in the Euclidean metric (see [1]). Therefore some additional assumptions on the coefficients is needed to ensure the regularity results. In [2–4], Campanato obtained the $L^{2,\lambda }$-regularity and Hölder continuity for the weak solutions of elliptic systems with continuous coefficients. See also [5–8] for related results.

Since the functions of vanishing mean oscillation (VMO) can have some kind of discontinuities, regularity results under a VMO assumption have been established by many authors; see, for example, [9–12] for elliptic systems, and [13–17] for subelliptic systems constructed by Hörmander’s vector fields. Huang in [9] established the gradient estimates in the generalized Morrey spaces of weak solutions to the linear elliptic systems with VMO coefficients. Similar results for the nonlinear elliptic systems were obtained by Daněček and Viszus in [10] and [11]. In [15] and [16] Di Fazio and Fanciullo proved that the local gradient estimates in [9] still hold true for the subelliptic systems structured on Hömander’s vector fields. Dong and Niu [14] established the Morrey and Campanato regularity for weak solutions to the nondiagonal subelliptic systems. The direct methods were mainly used to prove the desired results in the papers mentioned above. An important step of this kind of methods is to establish the higher integrability of gradients of weak solutions. These arguments were also used to prove the Morrey regularity and Hölder continuity for weak solutions to the obstacle problems associated with a single elliptic equation with constant coefficients or continuous coefficients; see [18–22].

Recently, another method called *A*-harmonic approximation has been widely applied to prove the optimal partial regularity for nonlinear elliptic systems or subelliptic systems in the Heisenberg group and Carnot groups; see [23–29]. This method is based on Simon’s technique of harmonic approximation ([30]) and generalized by Duzaar and Grotowski in [31] in order to deal with partial regularity for nonlinear elliptic systems. The key point is to show that a function which is “approximately harmonic”, i.e. a function closes sufficiently to some harmonic function in ${L^{2}}$. Making use of this method, one can simplify the proof avoiding the proof of a suitable reverse Hölder inequality for the gradient of a weak solution. We also mention that Daněček-John-Stará [32] proved the Morrey space regularity for weak solutions of Stokes systems with VMO coefficients by using a modified *A*-harmonic approximation lemma. Inspired by this work, Yu and Zheng [33] obtained optimal partial regularity for quasilinear elliptic systems with VMO coefficients by a modification of *A*-harmonic approximation argument.

In the present paper we study the interior regularity of weak solutions to the obstacle problem related to the system (1.1) by the technique of *A*-harmonic approximation, which implies that these solutions have the same kind of regularity as the weak solutions of (1.1). Throughout this article, we make the following assumptions.

1. The coefficients $a_{ij}^{\alpha \beta }$ are bounded measurable and such that, for some suitable $\lambda >0$ and $\Lambda >0$,
  $$ \lambda \vert \xi \vert ^{2}\leq a_{ij}^{\alpha \beta }(x) \xi _{\alpha }^{i}\xi _{\beta }^{j}\leq \Lambda \vert \xi \vert ^{2},\quad x\in \mathbb{R}^{n}, \xi \in \mathbb{R}^{mN}; $$
2. The functions ${B_{i}}$, $g_{i}^{\alpha }:\mathbb{R}^{n}\times \mathbb{R}^{N}\times \mathbb{R}^{mN}\rightarrow \mathbb{R}$ are both Carathéodory functions and for almost $x \in \Omega $ and all $(u,\xi ) \in \mathbb{R}^{N}\times \mathbb{R}^{mN}$, there exists $L>0$ such that
  $$ \bigl\vert B_{i} (x,u,\xi ) \bigr\vert \leq f_{i} (x)+L\vert \xi \vert ^{\gamma _{0}}, $$
  $$ \bigl\vert g_{i}^{\alpha }(x,u,\xi ) \bigr\vert \leq f_{i}^{\alpha }(x)+L\vert \xi \vert ^{\gamma }, $$
  where $1\leq \gamma _{0}<\frac{Q+2}{Q}$, $0\leq \gamma <1$, and
  $$ f\in L_{X}^{2Q/(Q+2),\lambda Q/(Q + 2)}\bigl(\Omega ,\mathbb{R}^{N} \bigr), \qquad \tilde{f} \in L_{X}^{2,\lambda } \bigl(\Omega , \mathbb{R}^{mN}\bigr), \quad Q- n< \lambda < Q. $$
  Here *Q* is the homogeneous dimension relative to Ω and $f=(f_{i} )$, $\tilde{f}=(f_{i}^{\alpha })$.

We are now in the position to state our main result.

### Theorem 1.1

*Suppose that* (H1)–(H2) *hold and that* $a_{ij}^{\alpha \beta }\in \operatorname{VMO}(\Omega )$ *for* $i,j=1,2,\ldots ,N$, $\alpha ,\beta =1,2,\ldots ,m$. *Let* $u\in \mathfrak{K}_{\psi }^{\theta }$ *be a weak solution to the obstacle problem for system* (1.1) *with* $X\psi \in L_{X}^{2,\lambda }(\Omega ,\mathbb{R}^{mN})$, *then* $Xu\in L_{X,\mathrm{loc}}^{2,\lambda } (\Omega ,\mathbb{R}^{mN})$. *Moreover*, *if* $Q - n<\lambda <2$ *then* $u \in C_{X}^{0,(2-\lambda )/2}(\Omega ,\mathbb{R}^{N})$.

The paper is organized as follows. In the next section we recall some concepts and facts associated to Carnot–Carathéodory spaces and give the proof of the modified *A*-harmonic approximation lemma for vector fields. In Sect. 3, we consider the following linear subelliptic system with VMO coefficients:

$$ {X^{\ast }} \bigl( {A(x)Xu} \bigr) ={X^{\ast }} \bigl( A(x)X\psi \bigr) , $$

and we prove a comparison principle and a Morrey type estimate for weak solutions of the above system by a modification of *A*-harmonic approximation argument. Section 4 is devoted to the proofs of Theorem 1.1. On the basis of the Morrey type estimate established for linear subelliptic system, we can first prove the $L_{X,\mathrm{loc}}^{2,\lambda }$-regularity for weak solutions of the obstacle problems and then interior Hölder continuity is obtained by virtue of the equivalence between the Campanato space and the Hölder continuity function space (see [34, 35]).

In what follows, we use *c* to denote a positive constant that may vary from line to line.

## Some notations and preliminaries

Let

$$ X_{\alpha }=\sum_{k=1}^{n}b_{\alpha k} \frac{\partial }{\partial x_{k}}, \quad b_{\alpha k}\in C^{\infty }, \alpha =1,2,\ldots ,m $$

be a family of vector fields in $\mathbb{R}^{n}$ satisfying Hörmander’s condition ([36]):

$$ \operatorname{rank} \bigl( {\operatorname{Lie}\{X_{1} ,\ldots,X_{m} \}}\bigr) =n. $$

We consider $X_{\alpha }$ as a first order differential operator acting on $u\in \text{Lip}(\mathbb{R}^{n})$ defined as

$$ X_{\alpha }u(x)= \bigl\langle {X_{\alpha }(x),\nabla u(x)} \bigr\rangle ,\quad \alpha =1,2,\ldots ,m. $$

We denote by $Xu=(X_{1} u,\ldots,X_{m}u)$ the gradient of *u* and hence $\vert Xu(x) \vert = ( \sum_{\alpha =1}^{m} \vert X_{\alpha }u(x) \vert ^{2} ) ^{\frac{1}{2}}$. An absolutely continuous curve $\gamma :[a,b]\to \mathbb{R}^{n}$ is said to be admissible if

$$ \gamma '(t)=\sum _{\alpha =1}^{m}{c_{\alpha }}(t)X_{\alpha } \bigl(\gamma (t)\bigr), \quad \text{a.e. } t\in [a,b], $$

for some functions $c_{\alpha }(t)$ satisfying $\sum_{\alpha =1}^{m} c_{\alpha }(t)^{2}\leq 1$. The Carnot–Carathéodory distance $d(x,y)$ generated by *X* is defined by

$$ d(x,y)=\inf \bigl\{ T>0:\mbox{there is an admissible curve} \gamma , \gamma (0)=x,\gamma (T)=y\bigr\} . $$

For $x\in \mathbb{R}^{n}$ and $R>0$ we let

$$ B_{R}(x)=B(x,R)=\bigl\{ y\in \mathbb{R}^{n}:d(y,x)< R\bigr\} . $$

In what follows, if $\sigma >0$ and $B=B(x,R)$ we write *σB* to indicate $B(x,\sigma R)$. Furthermore, if $E\subset \mathbb{R}^{n}$ is a Lebesgue measurable set with Lebesgue measure $\vert E \vert $, we set $u_{E}={⨍}_{E}u\;dx$ the integral average of *u* on *E*.

In [37], it was proved that for every connected $K\subset \Omega $ there exist constants $C_{1} ,C_{2} >0$ and $0<\lambda <1$ such that

$$ C_{1}\vert x - y \vert \leq d(x,y) \leq C_{2}\vert x - y \vert ^{\lambda }, \quad x,y\in K. $$

Moreover, there are $R_{d}>0$ and $C_{d}\geq 1$ such that, for any $x\in K$ and $R\leq R_{d} $,

2.1 $$ \bigl\vert B(x,2R) \bigr\vert \leq C_{d}\bigl\vert B(x,R) \bigr\vert . $$

Property (2.1) is the so-called “doubling condition” which is assumed to hold on the spaces of homogeneous type. The best constant $C_{d}$ in (2.1) is called the doubling constant. We call that $Q=\log _{2} C_{d} $ is the homogeneous dimension relative to Ω. As a consequence of (2.1), we have

2.2 $$ \vert B_{tR} \vert \geq C_{d}^{-2}t^{Q} \vert B_{R} \vert ,\quad \forall R \leq R_{d}, t \in (0,1). $$

We now introduce the relevant Sobolev spaces. Given $1\leq p<\infty $, the Sobolev space $S_{X}^{1,p}(\Omega ,\mathbb{R}^{N})$ is the Banach space

$$ S_{X}^{1,p}\bigl(\Omega ,\mathbb{R}^{N}\bigr)= \bigl\{ u\in L^{p}\bigl(\Omega ,\mathbb{R}^{N}\bigr): X_{\alpha }u\in L^{p}\bigl(\Omega ,\mathbb{R}^{N}\bigr),\alpha =1,2,\ldots ,m \bigr\} $$

endowed with the norm

$$ \Vert u \Vert _{S_{X}^{1,p}(\Omega ,\mathbb{R}^{N})}=\Vert u \Vert _{L^{p}(\Omega ,\mathbb{R}^{N})}+\sum_{\alpha =1}^{m}\Vert X_{\alpha }u \Vert _{L^{p}(\Omega ,\mathbb{R}^{N})}. $$

Here, $X_{\alpha }u$ is the distributional derivative of $u\in L_{\mathrm{loc}}^{1} (\Omega ,\mathbb{R}^{N})$ defined by

$$ \int _{\Omega }{X_{\alpha }}u \cdot \phi \,dx= \int _{\Omega }u\cdot X_{\alpha }^{\ast }\phi \,dx,\quad \forall \phi \in C_{0}^{\infty }\bigl(\Omega , \mathbb{R}^{N}\bigr), $$

where

$$ X_{\alpha }^{\ast }=-\sum _{k=1}^{n} \frac{\partial }{\partial x_{k}}(b_{\alpha k}\cdot ) $$

is the formal adjoint of $X_{\alpha }$, not necessarily a vector field in general. The space $S_{X,0}^{1,p} (\Omega ,\mathbb{R}^{N})$ is defined as the completion of $C_{0}^{\infty }(\Omega ,\mathbb{R}^{N})$ under the norm $\Vert \cdot \Vert _{S_{X}^{1,p}(\Omega ,\mathbb{R}^{N})}$.

In addition, we also need the following Sobolev inequalities for vector fields.

### Theorem 2.1

([38, 39])

*For every compact set* $K\subset \Omega $, *there exist constants* $C>0$ *and* $\bar{R}>0$ *such that*, *for any metric ball* $B=B(x_{0},R)$ *with* $x_{0}\in K$ *and* $0< R\leq \bar{R}$, *for any* $f\in S_{X}^{1,p}(B_{R})$,

$${\left({⨍}_{B_{R}}|f-f_{R}{|}^{\kappa p}\;dx\right)}^{\frac{1}{\kappa p}}\leq CR{\left({⨍}_{B_{R}}|Xf{|}^{p}\;dx\right)}^{\frac{1}{p}},$$

*where* $1\leq \kappa \leq Q/(Q-p)$, *if* $1\leq p< Q$; $1\leq \kappa <\infty $, *if* $p\geq Q$. *Moreover*,

$${\left({⨍}_{B_{R}}|f{|}^{\kappa p}\;dx\right)}^{\frac{1}{\kappa p}}\leq CR{\left({⨍}_{B_{R}}|Xf{|}^{p}\;dx\right)}^{\frac{1}{p}},$$

*whenever* $f \in S_{X,0}^{1,p}(B_{R})$.

Now we define the Morrey spaces, the Campanato spaces, VMO and the Hölder spaces with respect to the Carnot–Carathéodory metric. To simplify our description, we introduce the following notation:

$$ \Omega (x,R)=\Omega \cap B(x,R),\quad f_{x,R} =\frac{1}{\vert \Omega (x,R) \vert } \int _{\Omega (x,R)} f (y)\,dy, $$

and

$$ d_{0} =\min \{\operatorname{diam}\Omega ,R_{d}\}. $$

### Definition 2.2

For $1< p<\infty $ and $\lambda \leq Q$, we say that $f\in L_{\mathrm{loc}}^{p} (\Omega ,\mathbb{R}^{N})$ belongs to the Morrey space $L_{X}^{p,\lambda } (\Omega ,\mathbb{R}^{N})$ if

$$ \Vert f \Vert _{L_{X}^{p,\lambda }(\Omega ,\mathbb{R}^{N})} =\sup _{x\in \Omega ,0< \rho < d_{0}} \biggl( \frac{\rho ^{\lambda }}{\vert \Omega (x,\rho ) \vert } \int _{\Omega (x,\rho )}\bigl\vert f(y) \bigr\vert ^{p}\,dy \biggr) ^{\frac{1}{p}} < \infty ; $$

$f\in L_{\mathrm{loc}}^{p}(\Omega ,\mathbb{R}^{N})$ belongs to the Campanato space $\mathcal{L}_{X}^{p,\lambda }(\Omega ,\mathbb{R}^{N})$ if

$$ \Vert f \Vert _{\mathcal{L}_{X}^{p,\lambda }(\Omega ,\mathbb{R}^{N})} =\sup _{x\in \Omega ,0< \rho < d_{0}} \biggl( \frac{\rho ^{\lambda }}{\vert \Omega (x,\rho ) \vert } \int _{\Omega (x,\rho )}\bigl\vert f(y)-f_{x,\rho } \bigr\vert ^{p}\,dy \biggr) ^{\frac{1}{p}} < \infty . $$

### Definition 2.3

For $\alpha \in (0,1)$, the Hölder space $C_{X}^{0,\alpha }(\bar{\Omega },\mathbb{R}^{N})$ is the collection of functions $f:\bar{\Omega }\to \mathbb{R}^{N}$ satisfying

$$ \Vert f \Vert _{C_{X}^{0,\alpha }(\bar{\Omega },\mathbb{R}^{N})} =\sup _{\Omega }\vert f \vert + \sup _{\bar{\Omega }} \frac{\vert f(x)-f(y) \vert }{d(x,y)^{\alpha }}< \infty . $$

We say that *f* is locally Hölder continuous, i.e. $f\in C_{X}^{0,\alpha } (\Omega ,\mathbb{R}^{N})$, if $f\in C_{X}^{0,\alpha } (K,\mathbb{R}^{N})$ for every compact set $K\subset \Omega $.

### Definition 2.4

We say that $f\in L_{\mathrm{loc}}^{1} (\Omega ,\mathbb{R}^{N})$ belongs to $\mathrm{BMO}(\Omega ,\mathbb{R}^{N})$ if

$$ \Vert f \Vert _{\ast }=\sup _{x\in \Omega ,0< \rho < d_{0}} \frac{1}{\vert \Omega (x,\rho ) \vert } \int _{\Omega (x,\rho )}\bigl\vert f(y)-f_{x,\rho } \bigr\vert \,dy< \infty ; $$

*f* belongs to $\mathrm{VMO}(\Omega ,\mathbb{R}^{N})$ if $f\in {\mathrm{BMO}} (\Omega ,\mathbb{R}^{N})$ and

$$ \eta _{r}(f)=\sup _{x\in \Omega ,0< \rho < r}\frac{1}{\vert \Omega (x,\rho ) \vert } \int _{\Omega (x,\rho )}\bigl\vert f(y)-f_{x,\rho } \bigr\vert \,dy\to0, \quad r\to 0. $$

The integral characterization for a Hölder continuous function was shown in [35] and [34].

### Lemma 2.5

*If* $-p<\lambda <0$, *then* $\mathcal{L}_{X}^{p,\lambda }(\Omega ,\mathbb{R}^{N})\simeq C_{X}^{0,\alpha } (\Omega ,\mathbb{R}^{N})$, $\alpha =-\frac{\lambda }{p}$.

## Morrey type estimate for a subelliptic system

In this section we will prove by the modified *A*-harmonic approximation technique a Morrey type estimate for the subelliptic system

3.1 $$ {X^{\ast }} \bigl( {A(x)Xu} \bigr) = {X^{\ast }} \bigl( {A(x)X\psi } \bigr) . $$

Let us first recall that a function $h \in S_{X}^{1,2}(\Omega , \mathbb{R}^{N})$ is called *A*-harmonic for $A \in {\mathrm{Bil}}(\mathbb{R}^{mN})$ if *h* satisfies

$$ \int _{\Omega }{A(Xh,X\varphi )\,dx} = 0,\quad \forall \varphi \in C_{0}^{1}\bigl(\Omega ,\mathbb{R}^{N}\bigr). $$

We cite the *A*-harmonic approximation lemma for vector fields as follows ([24, 31]).

### Lemma 3.1

*Consider fixed positive* *λ* *and* Λ, *and* $m,N\in \mathbb{N}$ *with* $m\geq 2$. *Then for any given* $\varepsilon >0$ *there exists* $\delta =\delta (m,N,\lambda ,\Lambda ,\varepsilon )$ *with the following property*: *for any* $A\in {\mathrm{{Bil}}}(\mathbb{R}^{mN})$ *satisfying*

3.2 $$ A(\xi ,\xi ) \geq \lambda \vert \xi { \vert ^{2}}, \quad \textit{for all } \xi \in \mathbb{R}^{mN}, $$

*and*

3.3 $$ A(\xi ,\tilde{\xi })\leq \Lambda \vert \xi \vert \vert \tilde{\xi } \vert ,\quad \textit{for all } \xi ,\tilde{\xi }\in\mathbb{R}^{mN}, $$

*for any* $g\in S_{X}^{1,2}({B_{\rho }}(x_{0}),\mathbb{R}^{N})$ (*for some* $\rho >0$, ${x_{0}}\in \mathbb{R}^{n}$) *satisfying*

3.4 $${⨍}_{B_{\rho }(x_{0})}|Xg{|}^{2}\;dx\leq 1$$

*and*

3.5 $$|{⨍}_{B_{\rho }(x_{0})}A(Xg,X\varphi )\;dx|\leq \delta \underset{B_{\rho }(x_{0})}{\sup}|X\varphi |,\;\forall \varphi \in C_{0}^{\infty }\left(B_{\rho }(x_{0}),R^{N}\right),$$

*there exists an* *A*-*harmonic function* $h \in S_{X}^{1,2}(B_{\rho }(x_{0}),\mathbb{R}^{N})$ *such that*

$${⨍}_{B_{\rho }(x_{0})}|Xh{|}^{2}\;dx\leq 1\;\text{and}\;\frac{1}{\rho^{2}}{⨍}_{B_{\rho }(x_{0})}|g-h{|}^{2}\;dx\leq \varepsilon .$$

Similarly to [32] and [33], we can prove the following modification of the *A*-harmonic approximation lemma.

### Lemma 3.2

*Let* $0 < \lambda <\Lambda $ *and* $m\in \mathbb{N}$ *with* $m \geq 2$ *be fixed*. *Then*, *for any* $\varepsilon >0$, *there exists a constant* $k= k(m,N,\lambda ,\Lambda ,\varepsilon )$ *such that the following holds*: *for any* $A \in {\mathrm{{Bil}}}(\mathbb{R}^{mN})$ *satisfying conditions* (3.2), (3.3) *and for any* $u \in S_{X}^{1,2}(B_{\rho }(x_{0}),\mathbb{R}^{N})$, *there exists an* *A*-*harmonic function* $h \in S_{X}^{1,2}(B_{\rho }(x_{0}),\mathbb{R}^{N})$ *such that*

3.6 $$ \int _{B_{\rho }(x_{0})} \vert Xh \vert ^{2}\,dx \leq \int _{B_{\rho }(x_{0})}\vert Xu \vert ^{2}\,dx $$

*and*, *moreover*, *there exists* $\varphi \in C_{0}^{\infty }(B_{\rho }(x_{0}),\mathbb{R}^{N})$ *such that*

3.7 $$ \Vert X\varphi \Vert _{L^{\infty }(B_{\rho }(x_{0}),\mathbb{R}^{N})} \leq \frac{1}{\rho } $$

*and*

3.8 $$\begin{aligned} &\int _{B_{\rho }(x_{0})} {\vert u - h \vert ^{2}}\,dx \\ &\quad \leq \varepsilon {\rho ^{2}} \int _{B_{\rho }(x_{0})} \vert Xu \vert ^{2}\,dx + k(\varepsilon ) \biggl[ \frac{\rho ^{4}}{\vert B_{\rho }(x_{0}) \vert } \biggl( \int _{B_{\rho }(x_{0})} A(Xu,X\varphi )\,dx \biggr) ^{2} \biggr] . \end{aligned}$$

### Proof

For any given $\varepsilon > 0$ and $u \in S_{X}^{1,2}({B_{\rho }}({x_{0}}),\mathbb{R}^{N})$, we take $\delta (\varepsilon )$ as in the above Lemma 3.1 and set

$$g={\left({⨍}_{B_{\rho }(x_{0})}|Xu{|}^{2}\;dx\right)}^{-\frac{1}{2}}u.$$

Then (3.4) holds. Assume that for *g* the inequality (3.5) is true. From Lemma 3.1, there is an *A*-harmonic function *w* satisfying

$${⨍}_{B_{\rho }(x_{0})}|Xw{|}^{2}\;dx\leq 1,\;\frac{1}{\rho^{2}}{⨍}_{B_{\rho }(x_{0})}|w-g{|}^{2}\;dx\leq \varepsilon$$

and thus the function $h={\left({⨍}_{B_{\rho }(x_{0})}|Xu{|}^{2}\;dx\right)}^{\frac{1}{2}}w$ satisfies (3.6). Moreover, we have

$$|u-h{|}^{2}={⨍}_{B_{\rho }(x_{0})}|Xu{|}^{2}\;dx\cdot |g-w{|}^{2},$$

which implies

3.9 $$\int_{B_{\rho }(x_{0})}|u-h{|}^{2}\;dx\leq \int_{B_{\rho }(x_{0})}|Xu{|}^{2}\;dx\cdot {⨍}_{B_{\rho }(x_{0})}|g-w{|}^{2}\;dx\leq \varepsilon \rho^{2}\int_{B_{\rho }(x_{0})}|Xu{|}^{2}\;dx.$$

If, vice versa, there is a nonconstant function $\tilde{\varphi }\in C_{0}^{\infty }(B_{\rho }(x_{0}), \mathbb{R}^{N})$ such that

$$|{⨍}_{B_{\rho }(x_{0})}A(Xg,X\tilde{\varphi })\;dx|>\delta (\varepsilon )\underset{B_{\rho }(x_{0})}{\sup}|X\tilde{\varphi }|.$$

Setting $\varphi = \frac{\tilde{\varphi }}{\rho \sup _{B_{\rho }(x_{0})} \vert X\tilde{\varphi } \vert }$, it follows that

$$\frac{\rho }{\delta (\varepsilon )}|{⨍}_{B_{\rho }(x_{0})}A(Xg,X\varphi )\;dx|>1.$$

We now take $h = {u_{\rho }}$. Using the Poincaré inequality and the fact that $Xg={\left({⨍}_{B_{\rho }(x_{0})}|Xu{|}^{2}\;dx\right)}^{-\frac{1}{2}}Xu$ we deduce

3.10 $$\begin{array}{rl} \int_{B_{\rho }(x_{0})}|u-h{|}^{2}\;dx & =\int_{B_{\rho }(x_{0})}|u-u_{\rho }{|}^{2}\;dx\\ & \leq c\rho^{2}\int_{B_{\rho }(x_{0})}|Xu{|}^{2}\;dx\\ & \leq \frac{c\rho^{4}}{\delta^{2}(\varepsilon )}|{⨍}_{B_{\rho }(x_{0})}A(Xg,X\varphi )\;dx{|}^{2}\int_{B_{\rho }(x_{0})}|Xu{|}^{2}\;dx\\ & \leq \frac{c\rho^{4}|B_{\rho }(x_{0})|}{\delta^{2}(\varepsilon )}|{⨍}_{B_{\rho }(x_{0})}A(Xu,X\varphi )\;dx{|}^{2}\\ & \leq \frac{c\rho^{4}}{\delta^{2}(\varepsilon )|B_{\rho }(x_{0})|}|\int_{B_{\rho }(x_{0})}A(Xu,X\varphi )\;dx{|}^{2}. \end{array}$$

Combining (3.9) and (3.10) and taking $k(\varepsilon ) = \frac{c}{\delta ^{2} (\varepsilon ) }$ complete the proof. □

Now we are in a position to establish the Morrey type estimate for gradient of weak solution to (3.1) based on Lemma 3.2.

### Lemma 3.3

*Suppose that* $A(x)$ *satisfies* (H1) *and* $u \in S_{X,\mathrm{loc}}^{1,2}(\Omega , \mathbb{R}^{N})$ *is a weak solution to the system* (3.1), *i*.*e*.,

$$ \int _{\Omega }{A(x)} Xu \cdot X\varphi\,dx = \int _{\Omega }{A(x)} X\psi \cdot X\varphi \,dx,\quad \forall \varphi \in C_{0}^{\infty }\bigl(\Omega , \mathbb{R}^{N}\bigr). $$

*Then for any* ${x_{0}} \in \Omega $ *there exists a constant* $c > 0$ *such that*, *for all* $B_{\rho }(x_{0}) \subset B_{R} (x_{0}) \subset \Omega $, $R < {R_{d}}$,

3.11 $$ \int _{ B_{\rho }(x_{0})} \vert Xu \vert ^{2}\,dx \leq c \biggl[ \biggl( \frac{\rho }{R} \biggr) ^{Q} + \varepsilon + {\eta _{R}}(A) \biggr] \int _{ B_{R} (x_{0})} \vert Xu \vert ^{2}\,dx+ c \int _{ B_{R} (x_{0}) } \vert X\psi \vert ^{2}\,dx. $$

### Proof

For fixed ${x_{0}}\in \Omega $ and $0 < R < {R_{d}}$, denote ${B_{R}}: = {B_{R}}({x_{0}})$. Let *η* be a cut-off function on ${B_{R}}$ relative to ${B_{\rho }}$, i.e. $\eta \in C_{0}^{\infty }(B_{R}, \mathbb{R}^{N})$ and satisfies

$$ 0 \leq \eta (x) \leq 1, \qquad \eta (x) = 1 \quad \text{in } {B_{\rho }}, \qquad \bigl\vert X\eta (x) \bigr\vert \leq \frac{c}{R-\rho }. $$

Taking the function $\varphi ={\eta ^{2}}(u-u_{R})$ as a test function, it follows that

$$\begin{aligned} &\int _{B_{R}} {\eta ^{2}A(x)Xu \cdot Xu\,dx} \\ &\quad = - 2 \int _{B_{R}} {A(x)\eta (u - {u_{R}})Xu \cdot X\eta \,dx}+ \int _{B_{R}} \eta ^{2} A(x)X\psi \cdot Xu\,dx \\ &\quad \quad {}+ 2 \int _{B_{R}} {A(x)\eta (u - {u_{R}})X\psi \cdot X\eta \,dx}. \end{aligned}$$

From (H1) and Young’s inequality

$$\begin{aligned} \lambda \int _{B_{R}}\eta ^{2}\vert Xu \vert ^{2}\,dx&\leq 2\Lambda \int _{B_{R}}\vert \eta Xu \vert \vert u - {u_{R}} \vert \vert X\eta \vert \,dx \\ &\quad +\Lambda \int _{B_{R}} \vert \eta X\psi \vert \vert \eta Xu \vert \,dx + 2\Lambda \int _{B_{R}} {\vert \eta X\psi \vert \vert u - {u_{R}} \vert \vert X\eta \vert \,dx} \\ &\leq \varepsilon \int _{B_{R}} \eta ^{2}\vert Xu \vert ^{2}\,dx + \frac{c_{\varepsilon }}{(R-\rho )^{2}} \int _{B_{R}} \vert u - {u_{R}} \vert ^{2}\,dx + {c_{\varepsilon }} \int _{B_{R}} {\vert X\psi \vert ^{2}\,dx}. \end{aligned}$$

Choosing $\varepsilon <\lambda $, it follows that

3.12 $$ \int _{{B_{\rho }}} {\vert Xu{ \vert ^{2}}\,dx} \le \frac{c}{{{{(R - \rho )}^{2}}}} \int _{{B_{R}}} {\vert u - {u_{R}} { \vert ^{2}}\,dx} + c \int _{{B_{R}}} {\vert X\psi { \vert ^{2}}\,dx}. $$

Next we define $A_{R}={⨍}_{B_{R}}A(x)\;dx$. By Lemma 3.2, there exists an ${A_{R}}$-harmonic function $h \in S_{X}^{1,2}(B_{R}, \mathbb{R}^{N})$ such that (3.6)–(3.8) hold. Moreover, by standard results of the subelliptic system with constant coefficients (see for example [34]), we have

$$ \int _{B_{\rho }} {\vert Xh \vert ^{2}\,dx} \leq c \biggl( \frac{\rho }{R} \biggr) ^{Q} \int _{B_{R}} \vert Xh \vert ^{2}\,dx, \quad \forall 0 < \rho \leq R. $$

Therefore, from (3.12) and (3.6) it follows that for any $0 < \rho < R/2$

3.13 $$\begin{aligned} &\int _{B_{\rho }} {\vert Xu \vert ^{2}\,dx} \\ &\quad \leq \frac{c}{\rho ^{2}} \int _{B_{2\rho }} {\vert u - {u_{2\rho }} \vert ^{2}\,dx} + c \int _{B_{2\rho }} {\vert X\psi \vert ^{2}\,dx} \\ & \quad \leq \frac{c}{\rho ^{2}} \biggl( \int _{B_{2\rho }} \bigl\vert u-u_{2\rho }-(h- h_{2\rho }) \bigr\vert ^{2}\,dx + \int _{B_{2\rho }} \vert h - h_{2\rho } \vert ^{2}\,dx \biggr) + c \int _{B_{2\rho }} \vert X\psi \vert ^{2}\,dx \\ &\quad \leq \frac{c}{\rho ^{2}} \int _{B_{2\rho }} \vert u - h \vert ^{2}\,dx + c \int _{B_{2\rho }} \vert Xh \vert ^{2}\,dx + c \int _{B_{2\rho }} \vert X\psi \vert ^{2}\,dx \\ &\quad \leq \frac{c}{\rho ^{2}} \int _{B_{2\rho }}\vert u-h \vert ^{2}\,dx+ c \biggl( \frac{\rho }{R} \biggr) ^{Q} \int _{B_{R}}\vert Xh \vert ^{2}\,dx+c \int _{B_{R}}\vert X\psi \vert ^{2}\,dx \\ &\quad \leq \frac{c}{\rho ^{2}} \int _{B_{2\rho }} \vert u - h \vert ^{2}\,dx + c \biggl( \frac{\rho }{R} \biggr) ^{Q} \int _{B_{R}} {\vert Xu \vert ^{2}\,dx} + c \int _{B_{R}} {\vert X\psi \vert ^{2}\,dx}. \end{aligned}$$

For the first term in the right-hand side, we have from Lemma 3.2

3.14 $$\begin{aligned} \frac{c}{\rho ^{2}} \int _{B_{2\rho }}\vert u - h \vert ^{2}\,dx & \leq c \varepsilon \int _{B_{2\rho }}\vert Xu \vert ^{2}\,dx + ck( \varepsilon )\frac{\rho ^{2}}{\vert B_{2\rho } \vert } \biggl( \int _{B_{2\rho }} {A_{R}}Xu \cdot X\varphi\,dx \biggr) ^{2} \\ &\leq c\varepsilon \int _{B_{R}} \vert Xu \vert ^{2}\,dx + {c_{\varepsilon }}\frac{\rho ^{2}}{\vert B_{R} \vert } \biggl( \int _{B_{R}} {A_{R}}Xu \cdot X\varphi\,dx \biggr) ^{2}, \end{aligned}$$

where $\varphi \in C_{0}^{\infty }(B_{2\rho },\mathbb{R}^{N})$ satisfies $\Vert X\varphi \Vert _{L^{\infty }(B_{2\rho },\mathbb{R}^{N})} \leq \frac{1}{R}<\frac{1}{2\rho }$. Since *u* is a weak solution to (3.1), it follows that

$$\begin{aligned} \biggl(\int _{B_{R}} {A_{R}}Xu \cdot X\varphi\,dx \biggr)^{2}&= \biggl( \int _{B_{R}} ({A_{R}} - A)Xu \cdot X\varphi\,dx + \int _{B_{R}} AXu \cdot X\varphi\,dx \biggr) ^{2} \\ & \leq 2{ \biggl( { \int _{{B_{R}}} {({A_{R}} - A)Xu \cdot X\varphi \,dx} } \biggr) ^{2}} + 2{ \biggl( { \int _{{B_{R}}} {AXu \cdot X\varphi \,dx} } \biggr) ^{2}} \\ &: = {I_{1}} + {I_{2}}. \end{aligned}$$

From Hölder’s inequality, using (H1), we have

$$\begin{aligned} {I_{1}} &\leq \frac{1}{2\rho ^{2}} \int _{B_{R}} {\vert Xu \vert ^{2}\,dx} \cdot \int _{{B_{R}}} {\vert {A_{R}} - A \vert ^{2}\,dx} \\ & \leq \frac{\Lambda \vert B_{R} \vert }{\rho ^{2}}\frac{1}{\vert B_{R} \vert } \int _{B_{R}} {\vert {A_{R}} - A \vert \,dx} \cdot \int _{B_{R}} {\vert Xu \vert ^{2}\,dx} \\ &\leq \frac{\Lambda \vert B_{R} \vert }{\rho ^{2}}{\eta _{R}}(A) \int _{B_{R}} {\vert Xu \vert ^{2}\,dx} \end{aligned}$$

and

$$ {I_{2}} \leq \frac{\Lambda ^{2}}{2\rho ^{2}} \biggl( \int _{B_{R}} {\vert X\psi \vert \,dx} \biggr) ^{2} \leq \frac{\Lambda ^{2}\vert B_{R} \vert }{2\rho ^{2}} \int _{B_{R}} {\vert X\psi \vert ^{2}\,dx} . $$

Hence

3.15 $$ \biggl( \int _{B_{R}}{A_{R}}Xu \cdot X\varphi\,dx \biggr) ^{2} \leq \frac{c\vert {B_{R}} \vert }{\rho ^{2}} \biggl[ \eta _{R} (A) \int _{B_{R}} \vert Xu \vert ^{2}\,dx + \int _{B_{R}} \vert X\psi \vert ^{2}\,dx \biggr] . $$

Combining (3.15), (3.14) and (3.13), we have, for any $0 < \rho < R/2$,

$$ \int _{B_{\rho }} \vert Xu \vert ^{2}\,dx\leq c \biggl[ \biggl( \frac{\rho }{R} \biggr) ^{Q} + \varepsilon + {\eta _{R}}(A) \biggr] \int _{B_{R}}\vert Xu \vert ^{2}\,dx + c \int _{B_{R}} \vert X\psi \vert ^{2}\,dx . $$

For $R/2\leq \rho \leq R$, obviously

$$ \int _{B_{\rho }} {\vert Xu \vert ^{2}\,dx} \leq \int _{B_{R}} {\vert Xu \vert ^{2}\,dx} \leq {2^{Q}} { \biggl( \frac{\rho }{R} \biggr) ^{Q}} \int _{B_{R}} {\vert Xu \vert ^{2}\,dx}. $$

A combination of these two cases leads to (3.11) for $0 < \rho \le R$. □

We end this section with a comparison principle for system (3.1).

### Lemma 3.4

*Suppose that* $u,\psi \in S_{X}^{1,2} (B_{R}, \mathbb{R}^{N})$ *satisfy*

$$ {X^{\ast }} \bigl( {A(x)Xu} \bigr) = {X^{\ast }} \bigl( {A(x)X\psi } \bigr) $$

*where* $A(x)$ *satisfies* (H1). *If* $\psi \leq u$ *on* $\partial {B_{R}}$, *then* $\psi \leq u$ *a*.*e*. *in* ${B_{R}}$.

### Proof

For any $\varphi \in C_{0}^{\infty }(B_{R}, \mathbb{R}^{N})$ we have

3.16 $$ \int _{B_{R}} {A(x)Xu \cdot X\varphi \,dx} = \int _{B_{R}}A(x)X\psi \cdot X\varphi \,dx. $$

Set ${u_{+} }= \max \{ u,0\}$. Since $\psi \le u$ on $\partial {B_{R}}$, we conclude that (see [40, Lemma 6]) $(\psi -u)_{+} \in S_{X,0}^{1,2}({B_{R}}, \mathbb{R} ^{N})$ and

$$ X{(\psi - u)_{+}}= \textstyle\begin{cases} X(\psi - u),& \psi > u, \\ 0,& \psi \le u. \end{cases} $$

Choosing $\varphi = {(\psi - u)_{+} }$ in (3.16) gives

$$ \int _{B_{R}} {A(x)X(\psi - u) \cdot X{(\psi - u)}_{+}}\,dx = 0, $$

which implies

$$ \int _{B_{R}\cap \{ \psi > u\} } {A(x)X(\psi - u) \cdot X(\psi - u)\,dx} = 0. $$

From (H1) we have

$$\begin{aligned} \int _{B_{R}} \bigl\vert X{(\psi - u)_{+}} \bigr\vert ^{2}\,dx& = \int _{B_{R} \cap \{ \psi > u\} }\bigl\vert X(\psi - u) \bigr\vert ^{2}\,dx \\ &\leq \frac{1}{\lambda } \int _{B_{R} \cap \{\psi > u\}} A(x)X(\psi - u) \cdot X(\psi - u)\,dx=0. \end{aligned}$$

Thus from Poincaré inequality, we obtain

$$ \int _{B_{R}}\bigl\vert (\psi -u)_{+} \bigr\vert ^{2}\,dx \leq c{R^{2}} \int _{B_{R}}\bigl\vert X(\psi - u)_{+} \bigr\vert ^{2}\,dx = 0, $$

which implies ${(\psi - u)_{+} }=0$, or $\psi \leq u$ a.e. in ${B_{R}}$. The proof is complete. □

## Proof of main result

In this section we are going to prove our main result. To this end, we need a generalized iteration lemma, which can be found in [9, Proposition 2.1].

### Lemma 4.1

*Let* *H* *be a nonnegative almost increasing function on the interval* $[0,T]$ *and* *F* *a positive function on* $(0,T]$. *Suppose that*

1. *for any* $0<\rho \le R\le T$, *there exist* *A*, *B*, *ε* *and* $a>0$ *such that*
  $$ H(\rho )\leq \biggl( A \biggl( \frac{\rho }{R} \biggr) ^{a}+ \varepsilon \biggr) H(R)+BF(R); $$
2. *there exists* $\tau \in (0,a)$ *such that* $\frac{\rho ^{\tau }}{F(\rho )}$ *is almost increasing in* $(0,T]$. *Then there exist positive constants* $\varepsilon _{0} $ *and* *C* *such that*, *for any* $0\leq \varepsilon \leq \varepsilon _{0}$,
  $$ H(\rho )\leq C\frac{F(\rho )}{F(R)}H(R)+CB\cdot F(\rho ),\quad 0< \rho \leq R\leq T, $$
  *where* $\varepsilon _{0}$ *depends only on* *A*, *a* *and* *τ*.

### Proof of Theorem 1.1

Let $B_{R} =B(x_{0} ,R)\subset \subset \Omega $ be an arbitrary ball around $x_{0} $ of radius *R* and let $u\in \mathfrak{K}_{\psi }^{\theta }$ be a weak solution to the obstacle problem related to (1.1). In $B_{R}$ we split *u* as $u=w+(u-w)$, where $w\in S_{X}^{1,2} (B_{R},\mathbb{R}^{N})$ is the weak solution to the following system:

4.1 $$ \textstyle\begin{cases} X^{\ast } ( {A(x)Xw} ) =X^{\ast }( {A(x)X\psi } )& \mbox{in } B_{R}, \\ w=u&\mbox{on }\partial B_{R}. \end{cases} $$

Since $w=u\ge \psi $ a.e. on $\partial B_{R} $, it follows from Lemma 3.4 that $w\ge \psi $ a.e. in $B_{R} $.

By the definition of weak solutions, we have

$$ \int _{B_{R}} {A(x)} Xw\cdot X(w-u)\,dx= \int _{B_{R}} {A(x)} X\psi \cdot X(w-u)\,dx. $$

From (H1) and Young’s inequality one gets

$$\begin{aligned} \lambda \int _{B_{R} } \vert Xw \vert ^{2}\,dx &\leq \int _{B_{R}} {A(x)Xw\cdot Xw\,dx} \\ &\leq \Lambda \int _{B_{R}} \vert Xw \vert \vert Xu \vert \,dx +\Lambda \int _{B_{R}} \vert X\psi \vert \vert Xw-Xu \vert \,dx \\ &\leq \varepsilon \int _{B_{R}}\vert Xw \vert ^{2}\,dx +c_{\varepsilon } \int _{B_{R}} \vert Xu \vert ^{2}\,dx +c_{\varepsilon } \int _{B_{R}} \vert X\psi \vert ^{2}\,dx . \end{aligned}$$

Choosing $\varepsilon <\lambda $ leads to

4.2 $$ \int _{B_{R}} {\vert Xw \vert ^{2}\,dx} \leq c \int _{B_{R}} {\vert Xu \vert ^{2}\,dx} +c \int _{B_{R}} {\vert X\psi \vert ^{2}\,dx}. $$

On the basis of (4.2), it follows from Lemma 3.3 that for any $0<\rho \leq R$

4.3 $$\begin{aligned} & \int _{B_{\rho }} {\vert Xu \vert ^{2}\,dx} \\ &\quad \leq 2 \int _{B_{\rho }} {\vert Xw \vert ^{2}\,dx} +2 \int _{B_{\rho }} {\bigl\vert X(u-w) \bigr\vert ^{2}\,dx} \\ &\quad \leq c \biggl[ \biggl( \frac{\rho }{R} \biggr) ^{Q}+\varepsilon + \eta _{R} (A) \biggr] \int _{B_{R}} {\vert Xw \vert ^{2}\,dx} +c \int _{B_{R}} {\vert X\psi \vert ^{2}\,dx} +2 \int _{B_{\rho }} {\bigl\vert X(u-w) \bigr\vert ^{2}\,dx} \\ &\quad \leq c \biggl[ \biggl( \frac{\rho }{R} \biggr) ^{Q}+\varepsilon + \eta _{R} (A) \biggr] \int _{B_{R}} {\vert Xu \vert ^{2}\,dx} \\ &\quad \quad {}+c \int _{B_{R} } {\vert X\psi \vert ^{2}\,dx} +2 \int _{B_{R}} {\bigl\vert X(u-w) \bigr\vert ^{2}\,dx}. \end{aligned}$$

Note that $w-u$ is admissible as a test function in the definition of weak solutions to the obstacle problem due to $w-u\in S_{X,0}^{1,2} (B_{R},\mathbb{R}^{N})$ and $w\geq \psi $ a.e. in $B_{R}$. Applying $w-u$ to (1.2) leads to

$$ \int _{\Omega }{A(x)} Xu\cdot X(u-w)\,dx\leq \int _{\Omega }B(x,u,Xu) (u-w)\,dx+ \int _{\Omega }g (x,u,Xu)\cdot X(u-w)\,dx. $$

From (H1)–(H2) and Hölder’s inequality, we have

$$\begin{aligned} & \lambda\int _{B_{R}} {\vert Xu-Xw \vert ^{2}\,dx} \\ &\quad \leq \int _{B_{R}} {A(x)X(u-w)\cdot X(u-w)\,dx} \\ & \quad \leq{ -}\int _{B_{R}} {A(x)Xw\cdot X(u-w)\,dx} + \int _{B_{R}} {\bigl\vert B(x,u,Xu) \bigr\vert \vert u-w \vert \,dx} \\ & \quad \quad {} + \int _{B_{R}} {\bigl\vert g(x,u,Xu) \bigr\vert \vert Xu-Xw \vert \,dx} \\ &\quad \leq - \int _{B_{R} } {A(x)X\psi \cdot X(u-w)\,dx} + \int _{B_{R} } { \bigl( {\vert f \vert +L\vert Xu \vert ^{\gamma _{0} }} \bigr) \vert u-w \vert \,dx} \\ & \quad \quad {} + \int _{B_{R} } \bigl( \vert \tilde{f} \vert +L\vert Xu \vert ^{\gamma } \bigr) \vert Xu-Xw \vert \,dx \\ &\quad \leq c \biggl( \int _{B_{R}}\vert Xu-Xw \vert ^{2}\,dx \biggr) ^{\frac{1}{2}} \biggl[ \biggl( \int _{B_{R}} \vert X\psi \vert ^{2}\,dx \biggr) ^{\frac{1}{2}}+ \biggl( \int _{B_{R}} \bigl( \vert f \vert +\vert Xu \vert ^{\gamma _{0}} \bigr) ^{2Q/(Q+2)}\,dx \biggr) ^{\frac{Q+2}{2Q}} \biggr] \\ & \quad \quad {}+c \biggl( \int _{B_{R}}\vert Xu-Xw \vert ^{2}\,dx \biggr) ^{\frac{1}{2}} \biggl( \int _{B_{R}} \bigl( \vert \tilde{f} \vert +\vert Xu \vert ^{\gamma } \bigr) ^{2}\,dx \biggr) ^{\frac{1}{2}}, \end{aligned}$$

which means

4.4 $$\begin{aligned} \int _{B_{R}}\vert Xu-Xw \vert ^{2}\,dx &\leq c \int _{B_{R}} \vert X\psi \vert ^{2}\,dx+c \int _{B_{R}} \bigl( \vert \tilde{f} \vert +\vert Xu \vert ^{\gamma } \bigr) ^{2}\,dx \\ &\quad {} +c \biggl( \int _{B_{R}} \bigl( \vert f \vert +\vert Xu \vert ^{\gamma _{0}} \bigr) ^{2Q/(Q+2)}\,dx \biggr) ^{\frac{Q+2}{Q}}. \end{aligned}$$

In view of $1\leq \gamma _{0} <\frac{Q+2}{Q}$, $0\leq \gamma <1$, it follows by Hölder’s inequality that

4.5 $$\begin{aligned} \biggl( { \int _{B_{R}} {\vert Xu \vert ^{2\gamma _{0} Q/(Q+2)}\,dx}} \biggr) ^{\frac{Q+2}{Q}}&= \biggl[ { \biggl( { \int _{B_{R} } {\vert Xu \vert ^{2\gamma _{0} Q/(Q+2)}\,dx} } \biggr) ^{\frac{Q+2}{2\gamma _{0} Q}}} \biggr] ^{2\gamma _{0}} \\ &\leq \biggl[ {\vert B_{R} \vert ^{\frac{Q+2}{2\gamma _{0} Q}-\frac{1}{2}} \biggl( { \int _{B_{R} } {\vert Xu \vert ^{2}\,dx}} \biggr) ^{\frac{1}{2}}} \biggr] ^{2\gamma _{0}} \\ &=\vert B_{R} \vert ^{\frac{Q+2}{Q}-\gamma _{0} } \biggl( \int _{B_{R}}\vert Xu \vert ^{2}\,dx \biggr) ^{\gamma _{0}} \end{aligned}$$

and

4.6 $$\begin{aligned} \int _{B_{R}} {\vert Xu \vert ^{2\gamma }\,dx} &\leq \biggl[ \vert B_{R} \vert ^{\frac{1}{2\gamma } -\frac{1}{2}} \biggl( \int _{B_{R}}\vert Xu \vert ^{2}\,dx \biggr) ^{\frac{1}{2}} \biggr] ^{2\gamma } \\ & \leq \vert B_{R} \vert ^{1-\gamma } \biggl( \int _{B_{R}} \vert Xu \vert ^{2}\,dx \biggr) ^{\gamma } \\ & \leq \varepsilon \int _{B_{R}} {\vert Xu \vert ^{2}\,dx} +c_{\varepsilon }\vert B_{R} \vert . \end{aligned}$$

Combine (4.4)–(4.6) to deduce

4.7 $$\begin{aligned} \int _{B_{R}} \vert Xu-Xw \vert ^{2}\,dx &\leq c \bigl( \omega (R)+\varepsilon \bigr) \int _{B_{R}}\vert Xu \vert ^{2}\,dx+c \int _{B_{R}} \vert X\psi \vert ^{2}\,dx \\ &\quad +c \biggl( \int _{B_{R}}\vert f \vert ^{2Q/(Q+2)}\,dx \biggr) ^{\frac{Q+2}{Q}}+c \int _{B_{R}} \vert \tilde{f} \vert ^{2}\,dx+c_{\varepsilon } \vert B_{R} \vert , \end{aligned}$$

where $\omega (R)=\vert B_{R} \vert ^{\frac{Q+2}{Q}-\gamma _{0}} ( \int _{B_{R}}\vert Xu \vert ^{2}\,dx ) ^{\gamma _{0}-1}$.

From (4.7) and (4.3), we find, for any $0<\rho \leq R$ (we may suppose $R<1$),

$$\begin{aligned} \int _{B_{\rho }} {\vert Xu \vert ^{2}\,dx} &\leq c \biggl[ { \biggl( {\frac{\rho }{R}} \biggr) ^{Q}+\varepsilon +\eta _{R} (A)+\omega (R)} \biggr] \int _{B_{R} } {\vert Xu \vert ^{2}\,dx} +c \int _{B_{R} } {\vert X\psi \vert ^{2}\,dx} \\ &\quad +c \biggl( { \int _{B_{R}} {\vert f \vert ^{2Q/(Q+2)}\,dx}} \biggr) ^{(Q+2)/Q}+c \int _{B_{R}} \vert \tilde{f} \vert ^{2}\,dx +c \vert B_{R} \vert \\ &\leq c \biggl[ { \biggl( {\frac{\rho }{R}} \biggr) ^{Q}+\vartheta (R)} \biggr] \int _{B_{R} } {\vert Xu \vert ^{2}\,dx}+\tilde{c} \frac{\vert B_{R} \vert }{R^{\lambda }}, \end{aligned}$$

where $\vartheta (R)=\varepsilon +\eta _{R} (A)+\omega (R)$, $\tilde{c}=\tilde{c}(\Vert f \Vert _{L^{2Q/(Q+2),\lambda Q/(Q+2)}}^{2}+\Vert \tilde{f} \Vert _{L^{2,\lambda }}^{2} +\Vert X\psi \Vert _{L^{p,\lambda }}^{2})$. By the absolute continuity of Lebesgue integral, we know that $\omega (R)\to 0$ as $R\to 0$. Finally, we can take $R< R_{0} $ such that $\eta _{R}(A)$ is small enough due to the VMO property of $A(x)$. If we take $F(\rho )=\frac{\vert B_{\rho } \vert }{\rho ^{\lambda }}$, $0< Q-\lambda <\tau <Q$, we claim that $\frac{\rho ^{\tau }}{F(\rho )}=\frac{\rho ^{\tau +\lambda }}{\vert B_{\rho } \vert }$ is almost increasing. In fact, it follows from (2.2) that, for any $t\in (0,1)$,

$$ \frac{(t\rho )^{\tau }}{F(t\rho )}/\frac{\rho ^{\tau }}{F(\rho )}=\frac{(t\rho )^{\tau +\lambda }}{\vert B_{t\rho } \vert }/\frac{\rho ^{\tau +\lambda }}{\vert B_{\rho } \vert }= \frac{t^{\tau +\lambda }\vert B_{\rho } \vert }{\vert B_{t\rho } \vert }\leq C_{d}^{2} t^{\tau +\lambda -Q}\le C_{d}^{2}. $$

By Lemma 4.1, we obtain, for $0<\rho \leq R$,

4.8 $$ \int _{B_{\rho }} {\vert Xu \vert ^{2}\,dx} \leq c \frac{\vert B_{\rho } \vert }{\rho ^{\lambda }}, $$

which shows that $Xu\in L_{X,\mathrm{loc}}^{2,\lambda } (\Omega ,\mathbb{R}^{mN} )$.

On the other hand, from Poincaré inequality and (4.8) we see that

$$\begin{aligned} &\quad \int _{B_{\rho }}\vert u-u_{\rho } \vert ^{2}\,dx \leq c\rho ^{2} \int _{B_{\rho }} \vert Xu \vert ^{2}\,dx\leq c \frac{\vert B_{\rho } \vert }{\rho ^{\lambda -2}}, \end{aligned}$$

which implies $u\in {\mathcal{L}}_{X,\mathrm{loc}}^{2,\lambda -2} (\Omega ,\mathbb{R}^{N})$ and so $u \in C_{X}^{0,(2-\lambda )/2}(\Omega ,\mathbb{R}^{N})$ according to Lemma 2.5. The proof is finished. □
